# Cytokines and Chemokines Involved in Osteoarthritis Pathogenesis

**DOI:** 10.3390/ijms22179208

**Published:** 2021-08-26

**Authors:** Vilim Molnar, Vid Matišić, Ivan Kodvanj, Roko Bjelica, Željko Jeleč, Damir Hudetz, Eduard Rod, Fabijan Čukelj, Trpimir Vrdoljak, Dinko Vidović, Mario Starešinić, Srećko Sabalić, Borut Dobričić, Tadija Petrović, Darko Antičević, Igor Borić, Rok Košir, Uršula Prosenc Zmrzljak, Dragan Primorac

**Affiliations:** 1 St. Catherine Specialty Hospital, 49210 Zabok, Croatia; vilim.molnar@svkatarina.hr (V.M.); vid.matisic@svkatarina.hr (V.M.); roko.bjelica@gmail.com (R.B.); zeljko.jelec@svkatarina.hr (Ž.J.); ortohud@gmail.com (D.H.); eduard.rod@svkatarina.hr (E.R.); fabijan.cukelj@svkatarina.hr (F.Č.); trpimir.vrdoljak@svkatarina.hr (T.V.); dinko.vidovic@gmail.com (D.V.); dobricic_borut@yahoo.de (B.D.); tadijap@gmail.com (T.P.); darko.anticevic@gmail.com (D.A.); igor.boric@svkatarina.hr (I.B.); 2 St. Catherine Specialty Hospital, 10000 Zagreb, Croatia; 3 Faculty of Medicine, Josip Juraj Strossmayer University of Osijek, 31000 Osijek, Croatia; 4 Department of Pharmacology, School of Medicine, University of Zagreb, 10000 Zagreb, Croatia; ikodvanj@gmail.com; 5 Department of Nursing, University North, 48000 Varaždin, Croatia; 6 Department of Orthopaedic Surgery, Clinical Hospital “Sveti Duh”, 10000 Zagreb, Croatia; 7 University Hospital “Sisters of Mercy”, Clinic for Traumatology, Draškovićeva 19, 10000 Zagreb, Croatia; ssabalic@gmail.com; 8 Department of Health Studies, University of Split, 21000 Split, Croatia; 9 Department of Traumatology, Medical University Merkur Hospital, 10000 Zagreb, Croatia; 10 Medical School, University of Split, 21000 Split, Croatia; mstaresinic@yahoo.com; 11 Department of Orthopaedics and Traumatology, University Hospital Dubrava, 10000 Zagreb, Croatia; 12 Faculty of Dental Medicine and Health, Josip Juraj Strossmayer University of Osijek, 31000 Osijek, Croatia; 13 Medical School, University of Mostar, 88000 Mostar, Bosnia and Herzegovina; 14 Medical School, University of Rijeka, 51000 Rijeka, Croatia; 15 Molecular Biology Laboratory, BIA Separations CRO, Labena Ltd., 1000 Ljubljana, Slovenia; rok.kosir@labena.si (R.K.); ursula.prosenc@biaseparationscro.com (U.P.Z.); 16 Medical School REGIOMED, 96450 Coburg, Germany; 17 Eberly College of Science, State College, The Pennsylvania State University, University Park, PA 16802, USA; 18 The Henry C. Lee College of Criminal Justice and Forensic Sciences, University of New Haven, West Haven, CT 06516, USA

**Keywords:** osteoarthritis, cytokines, chemokines, pathogenesis, inflammation, biomarker

## Abstract

Osteoarthritis is a common cause of disability worldwide. Although commonly referred to as a disease of the joint cartilage, osteoarthritis affects all joint tissues equally. The pathogenesis of this degenerative process is not completely understood; however, a low-grade inflammation leading to an imbalance between anabolic and katabolic processes is a well-established factor. The complex network of cytokines regulating these processes and cell communication has a central role in the development and progression of osteoarthritis. Concentrations of both proinflammatory and anti-inflammatory cytokines were found to be altered depending on the osteoarthritis stage and activity. In this review, we analyzed individual cytokines involved in the immune processes with an emphasis on their function in osteoarthritis.

## 1. Introduction

Osteoarthritis (OA) is the most common musculoskeletal condition and the largest cause of disability in the world [1]. The knee is predominantly affected in OA. A recent study concluded that knee OA globally affects 16% of the population, more often women, and that its prevalence, due to today’s lifestyle, higher obesity rates and higher average life expectancy, is constantly increasing [2]. Although OA is often referred to as a joint disease with damage and loss of cartilage, OA is a much more diverse disease with complex pathogenesis that affects all tissues within the joint [3].

**Figure 1:**
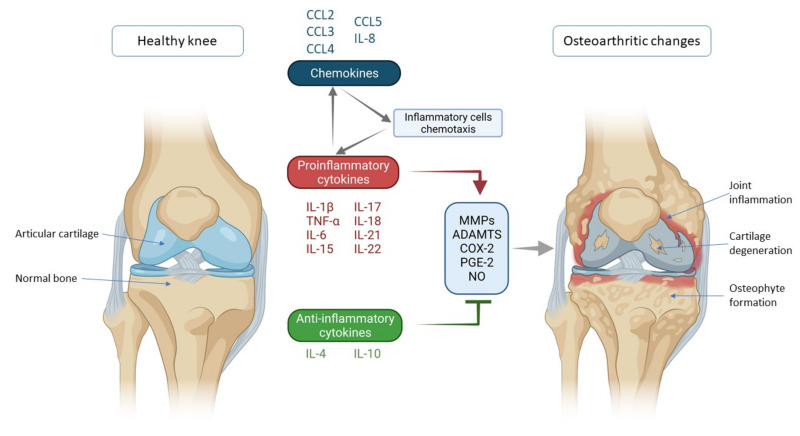
Schematic representation of key inflammatory processes and factors in osteoarthritis pathogenesis. The disturbed balance of proinflammatory and anti-inflammatory cytokines (in favor of proinflammatory cytokines) is responsible for the secretion of enzymes and other inflammatory factors involved in the pathogenesis of osteoarthritis leading to morphological changes within the joint such as cartilage degeneration, osteophyte formation and other inflammatory changes such as synovitis. Chemokines also contribute to inflammatory processes, stimulating the chemotaxis of inflammatory cells that then further secrete proinflammatory cytokines, thus creating a vicious circle that poses a major challenge in treating and slowing the progression of osteoarthritis. IL—interleukin; CCL-CC—chemokine ligand; TNF-α—tumor necrosis factor α; MMPs—matrix metalloproteinases (MMPs); ADAMTS—a disintegrin-like and metalloproteinase with thrombospondin motif; COX-2—cyclooxygenase-2; PGE-2—prostaglandin E2; NO—nitric oxide.

One of the most important factors in the pathogenesis of OA is a disturbed cytokine balance in favor of proinflammatory cytokines that by their action initiate a vicious cycle that leads to final effects such as damage to cartilage and other intra-articular structures by activating catabolic enzymes (matrix metalloproteinases (MMPs) and ADAMTS (a disintegrin-like and metalloproteinase with thrombospondin motif)) (Figure 1) [4]. The most important inflammatory mediators in the pathogenesis of OA are IL-1β, TNF-α and IL-6. They are activators of a plethora of different signaling pathways that activate other cytokines and pathologic processes. Part of this unstoppable process are chemokines that, stimulated by cytokines, attract inflammatory cells to the joint that further promote the secretion of inflammatory factors and disease progression [5]. The aim of this review was to describe the mechanisms of action of the most important cytokines and chemokines involved in OA pathogenesis, with emphasis on knee OA.

## 2. Cytokines and Chemokines Involved in Knee Osteoarthritis Pathogenesis

### 2.1. Proinflammatory Cytokines

#### 2.1.1. IL-1β

IL-1β is one of the main proinflammatory cytokines involved in the pathogenesis of numerous diseases and a member of the IL-1 superfamily, which consists of IL-1α, IL-1β, IL36α, IL-36β, IL-36γ, IL-36RA, IL-37, IL-38 and IL-1Ra (IL-1 receptor antagonist). It achieves its effects by binding to the receptor named type I IL-1 receptor I (IL-1RI), a type I transmembrane protein that is the binding site of IL-1α and IL-1Ra as well [6]. IL-1Ra competes for an IL-1RI binding site with IL-1β with antagonistic activity. These receptors are expressed on a number of cell types in the knee joint, including chondrocytes, synoviocytes, osteoblasts, osteoclasts and inflammatory cells such as macrophages [7]. Furthermore, it has been observed that the number of IL-1RI is increased in isolated human OA chondrocytes in vitro [8]. By binding to the receptor, IL-1β activates several signaling pathways, which, combined, lead to the progression of OA. IL-1Ra binds to the same receptors as IL-1β and acts as its competitive antagonist, thus blocking IL-1β proinflammatory effects. Although IL-1Ra is an anti-inflammatory mediator, its plasma levels have been found to correlate with the radiological stage of symptomatic OA and its progression, regardless of risk factors such as age, sex and body mass index, confirming the idea of a constant competition of proinflammatory and anti-inflammatory factors in OA [9].

Through mitogen-activated protein kinase (MAPK) signaling, IL-1β induces catabolic events such as cartilage degradation, as the most dominant process in OA. MAPK consists of three families: extracellular signal-regulated kinases (ERKs), c-Jun N-terminal kinases (JNKs) and p38 MAPKs. By downregulating type II collagen and aggrecan gene expression, ERK activation by IL-1β reduces cartilage extracellular matrix (ECM) production [10]. JNK signaling also inhibits collagen synthesis through SOX-9 suppression [11]. Furthermore, IL-1β leads to ECM degradation by inducing collagenases and aggrecanases such as MMP-1 (via ERK, p38, JNK), MMP-3 (via ERK), MMP-13 (via ERK, p38, JNK), ADAMTS-4 (via ERK, p38, JNK) and ADAMTS-5 (via JNK) [12]. These catabolic events result in chondrocyte hypertrophy, dedifferentiation and, finally, apoptosis [13]. Through all three MAPK signaling pathways, IL-1β stimulates the secretion of IL-6, LIF and other proinflammatory cytokines, which potentiate the catabolic effects of IL-1β and at the same time serve as catabolic mediators on their own [12]. In that way, IL-1β can upregulate itself through a positive feedback mechanism. ERK-mediated effects can also be activated by PGE-2 (prostaglandin E2), NO (nitric oxide) and COX-2 (cyclooxygenase-2), inflammatory mediators that are, again, induced by IL-1β [14]. These mediators also contribute to synovial inflammation, which additionally enhances the secretion of IL-1β and other cytokines and aggravates the vicious circle of OA progression [15].

Another important signaling pathway in IL-1β mediated OA progression is NF-κB, which, when activated, leads to inhibition of type II collagen expression, increased production of matrix metalloproteinases (MMP-1, MMP-2, MMP-3, MMP-7, MMP-8, MMP-9 and MMP-13) and aggrecanases (ADAMTS4 and ADAMTS5), but also COX-2, iNOS, PGE-2 and NO [16,17]. Additionally, the IL-1β-activated NF-κB pathway supports proinflammatory cytokines synthesis and secretion, such as IL-6 and TNF-α [16].

**Figure 2:**
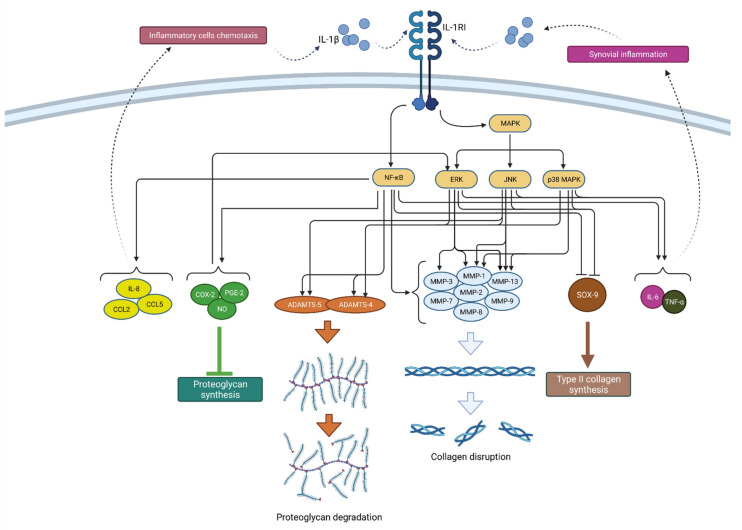
Schematic representation of IL-1β function in osteoarthritis pathogenesis. By binding to its receptor (IL-1RI), IL-1β activates signaling pathways (NF-κB and MAPK) that, by raising the expression of enzymes (ADAMTS and MMPs), lead to catabolic reactions, i.e., proteoglycan degradation and collagen disruption. Furthermore, via the same signaling pathways, IL-1β inhibits type II collagen synthesis through SOX-9 suppression but also proteoglycan synthesis by increasing the synthesis of COX-2, PGE-2 and NO. In addition, IL-1β increases the expression of chemokines such as IL-8, CCL2 and CCL5, as well as the cytokines IL-6 and TNF-α, which attract inflammatory cells and cause synovial inflammation, respectively, resulting in the even greater production and secretion of IL-1β. IL-1β—interleukin 1β; IL-1RI—interleukin 1 receptor 1; MAPK—mitogen-activated protein kinase; ERK—extracellular signal-regulated kinases; JNK—c-Jun N-terminal kinases; NF-κB—nuclear factor kappa-light-chain-enhancer of activated B cells; MMPs—matrix metalloproteinases (MMPs); ADAMTS—a disintegrin-like and metalloproteinase with thrombospondin motif; COX-2—cyclooxygenase-2; PGE—prostaglandin E2; NO—nitric oxide; IL-8—interleukin 8; CCL2—chemokine ligand 2; CCL—chemokine ligand 5; SOX-9—SRY-Box Transcription Factor 9; IL-6—interleukin 6; TNF-α—tumor necrosis factor α.

Furthermore, IL-1β-mediated NF-κB activation stimulates the production of various chemokines including IL-8, monocyte chemoattractant protein-1 (MCP-1 or CCL2), CCL5, also known as RANTES (regulated on activation, normal T cell expressed and secreted) and macrophage inflammatory protein-1a (MIP-1a), which, by attracting additional inflammatory cells, potentiate the inflammatory state in the joint [4]. In addition, activated macrophages, attracted to the synovial tissue due to the effects of chemokines, are the primary source of IL-1β secretion in the synovium, which once again confirms the complexity of the vicious inflammatory cycle in OA [12]. A schematic representation of the mechanism of action and effects of IL-1β is shown in Figure 2.

Due to its significant proinflammatory effects and ability to activate a number of signaling pathways in the pathogenesis of OA, the suppression of IL-1β action has been studied as a potential therapeutic method in treating OA and stopping its progression. However, IL-1β inhibition did not produce the desired effects of preventing OA progression; therefore, the negative results led to the idea that IL-1β does not likely drive OA progression [18,19,20,21]. With that in mind, researchers should consider that the pathogenesis of OA does not depend on a single cytokine; rather, the same signaling pathways can be activated by different cytokines, and the interplay of multiple factors is crucial in the onset and progression of the disease.

#### 2.1.2. TNF-α

TNF-α is a potent proinflammatory cytokine that plays an important role in the inflammatory response. As such, it is involved in cell differentiation, proliferation and apoptosis [22]. TNF-α was discovered in 1975 by Carswell et al. as a protein that showed cytotoxic activity and caused the necrotic regression of certain tumor types. Alongside IL-1β, this cytokine is considered the key proinflammatory cytokine in the pathogenesis of OA [23].

**Figure 3:**
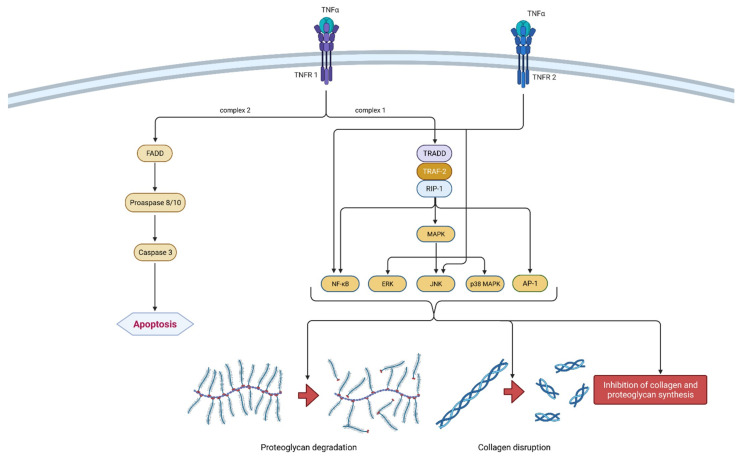
Schematic representation of TNF-α function in osteoarthritis pathogenesis. TNF-α can bind to two receptors, TNRF-1 and TNRF-2. By binding to TNRF-1, TNF-α can induce two different signaling complexes. Complex 1 leads to the stimulation of cell survival and the expression of NF-κB, MAPK and AP-1, which results in proteoglycan degradation, collagen disruption and the inhibition of proteoglycan and collagen synthesis. On the other hand, the activation of complex 2 leads to a cascade of reactions, which include the formation of FADD and the activation of procaspase 8/10 and caspase 3, which consequently leads to cell apoptosis. Additionally, the binding of TNF-α to TNRF-2 activates NF-κB and JNK. In summation, TNF-α leads to degeneration of cartilage and other joint structures, thus contributing to the onset and progression of osteoarthritis. TNF-α—tumor necrosis factor α; TNRF-1—Tumor necrosis factor receptor 1; TNRF-2—Tumor necrosis factor receptor 2; TRADD—TNFR-1 associated death domain protein; RIP-1—receptor interacting protein-1; TRAF-2—TNF receptor-associated factor-2; MAPK—mitogen-activated protein kinase; ERK—extracellular signal-regulated kinases; JNK—c-Jun N-terminal kinases; NF-κB—nuclear factor kappa-light-chain-enhancer of activated B cells; AP-1—activator protein 1; FADD—Fas-associated death domain protein.

It is a part of the tumor necrosis factors superfamily, together with 18 other ligands [24]. The TNF superfamily members are type II transmembrane proteins that can be expressed in soluble and membrane-bound forms [25]. TNF-α is a homotrimeric, cone-shaped protein secreted in two forms, as mentioned above. The membrane-bound form (tmTNF-α) differs from the soluble form (sTNF-α) in its biological activity and is considered more active [26]. TNF-α binds to two isotypes of membrane receptors present on almost all known cell types except erythrocytes and unstimulated T lymphocytes. Tumor necrosis factor receptor 1 (TNRF-1) can be activated by both TNF-α forms, while TNRF-2 is mainly activated by the membrane form. Westacott et al. claim that TNRF-1 activity has a greater impact on local cartilaginous tissue loss, but both receptors are involved in signal transduction related to the pathogenesis of OA [27]. Due to their differences and unique structural features, both receptors are able to participate in different signal pathways [28]. Ligands can induce two different signaling complexes by binding to TNRF-1 receptors. Complex 1 leads to the stimulation of cell survival and the expression of pro-inflammatory genes and complex 2 leads to apoptosis and cell death. Complex 1 is associated with TNFR-1 associated death domain protein (TRADD), which allows for the binding of another two adapter proteins—receptor interacting protein-1 (RIP-1) and TNF receptor-associated factor-2 (TRAF-2). The most important transcription pathways are NF-κB and AP-1. Furthermore, another important signaling pathway is activated by mitogen-activated protein kinases (MAPK), more precisely by its three independent pathways (ERK, JNK and p38 MAPK). On the contrary, signaling complex 2 is directed towards cell death or apoptosis [28,29]. The formation of FADD (Fas-associated death domain protein), procaspase 8/10 and caspase 3 are responsible for programmed cell death. Not so long ago, TNRF-2 initiated signaling was considered less investigated than those initiated by the activation of TNRF-1 receptors. It is claimed that TNRF-2 stimulation notably supports cell activation, migration and proliferation [29]. It activates the JNK kinase and the transcription factor NF-κB. It is worth mentioning that polymorphism in the gene (*M196R*) encoding TNFR-2 may predetermine the development of OA by increasing the number of receptor proteins on the surface of chondrocytes [28]. The mechanism of action of TNF-α is shown in Figure 3.

The activation of the same signaling pathways as IL-1ß results in synergism between these two cytokines [30]. Chondrocytes’ synthesis of proteoglycan components and type II collagen is blocked by TNF-α [31]. TNF-α also leads to extracellular matrix (ECM) degradation by inducing collagenases and aggrecanases including MMP-1, MMP-3, MMP-13 and ADAMTS-4, which coincides with IL-1β [32]. The possibility of cartilage repair is vastly reduced because of the earlier mentioned complex 2 signaling pathway and consequent cell apoptosis. Furthermore, TNF-α increases the synthesis of IL-6, IL-8, RANTES and VEGF. Together with the already mentioned IL-1β, TNF-α induces the production of iNOS, COX-2 and PGE-2 synthase, which further upregulates IL-1β and TNF-α production [28]. Considering its proinflammatory nature, it is important to mention that the inhibition of TNF-α could be a sufficient therapeutic option in treating OA. Present data suggest that monoclonal antibodies may exhibit a favorable risk-benefit ratio considering future targeted therapeutic methods. However, current monoclonal antibodies targeting TNF-α such as adalimumab, infliximab and etanercept have shown poor results in clinical studies of general OA patients. They demonstrated only limited benefits in pain reduction and no significant disease modification [33].

#### 2.1.3. IL-6

Classic immunology textbooks commonly depict interleukin-6 (IL-6) as a proinflammatory cytokine important in many inflammatory diseases [34,35]. Contrary to this, the biological role of IL-6 is far more complex.

In 1986, the molecular cloning and structural analysis of B-cell differentiation factor (BCDF) was first reported [36]. Today, BCDF is known as interleukin-6, and as its name suggests, IL-6 is a protein that is essential for the communication between leukocyte cells [37]. IL-6 is a member of the IL-6 family (IL-11, ciliary neurotrophic factor, leukemia inhibitory factor, oncostatin M, cardiotrophin 1, cardiotrophin-like cytokine and IL-27), a group of cytokines that share a common signal-transducing protein gp130 that signals through various signaling pathways, including JAK/STAT (Janus kinase/signal transducers and activators of transcription) and MAPK, PI3K (phosphoinositide 3-kinases), and to which IL-6 has no binding affinity [38,39]. Although it is most commonly mentioned in the context of immune system functioning, IL-6 is essential for various organ systems, including the hematopoietic, endocrine and nervous system, and it is classified as an adipokine and myokine [40,41]. It is produced by a number of cells, including T cells, B cells, granulocytes, smooth muscle cells, eosinophils, mast cells, glial cells and keratinocytes, but in the context of OA, chondrocytes, osteoblasts and synoviocytes are the most important to mention [42,43].

IL-6 acts by binding to the IL-6 receptors, either membrane-bound (mbIL-6R) or soluble (sIL-6R). The binding of IL-6 to sIL-6R forms a complex that associates with ubiquitously expressed gp130 protein and activates *trans*-signaling responsible for the proinflammatory action of IL-6 [44]. On the other hand, the binding of IL-6 and selectively expressed mbIL-6R is considered to form a complex that activates *classic*-signaling responsible for the anti-inflammatory and regenerative properties of IL-6 [44]. Trans-signaling affects virtually all cell types since gp130 is ubiquitously expressed, while classic signaling only affects cells that express mbIL-6R, namely, the hepatocytes, neutrophils, monocytes, macrophages, osteocytes, chondrocytes and some lymphocytes [45,46,47]. The concentration of sIL-6R is considered a determining factor of *trans-* or classic signaling dominance [48,49]. sIL-6R is considered to be produced as a result of the alternative splicing of mRNA and, to a greater extent, by the proteolytic cleavage of mbIL-6R mediated by metzincin type proteases that are known to have increased expression in OA, specifically a disintegrin and metalloproteinases 10 and 17 (ADAM10 and ADAM17) [50,51]. Furthermore, the endogenous soluble form of gp130 (sgp130) has the ability to bind and stabilize IL-6 and sIL-6R. Although it was initially claimed that this could suppress *trans*-signaling without affecting the classic signaling, newer studies have shown that inhibition of classic signaling can occur as well when there is a molar excess of sIL-6R over IL-6 [52,53,54]. Interestingly, ADAM10 and 17 can also shed membrane-bound gp130, but their affinity for gp130 is small and thus their proteolytic activity against gp130 is likely not biologically significant [55].

IL-6’s exact role in OA is difficult to define, as there are beneficial and detrimental effects of IL-6. In vitro studies on chondrocytes have shown that IL-6 alone can induce TIMP-1, with the effect even more pronounced when chondrocytes are co-treated with sIL-6R [56,57]. Some studies have shown that IL-6, with and without sIL-6R, increases the expression of collagen type 2, while others have shown that IL-6 or IL-6 + sIL-6R treatment inhibits collagen type 2 production via transcriptional control [58]. The combination of IL-6 and sIL-6R induces the expression of MMP-1, 3 and 13 and ADAMTS-4, 5/11 [57]. On the other hand, animal studies have shown that IL-6 knockout mice develop OA in higher prevalence and severity than wild-type mice and that IL-6 intraarticular injection induces OA-like cartilage lesions [45,59]. Conversely, the systemic treatment using anti-IL-6 or STAT-3 alleviated experimental OA in mice [57].

When compared with healthy controls, patients with end-stage OA have a significantly higher concentration of IL-6 in synovial fluid (median 4.8 vs. 196.9 pg/mL), and the concentration of IL-6 in synovial fluid is known to correlate with the pain experienced by patients with OA [60,61]. Additionally, IL-6 seems to have a predictive value as well. In a prospective cohort study conducted on 163 subjects aged 50–79, disease severity assessment and IL-6 and TNF-α serum measurements were performed at baseline and a 3-year follow-up. The findings of both univariate and multivariate analyses suggest that increased IL-6 and TNF-α concentration at baseline are associated with an increased loss of cartilage volume [62]. Furthermore, the decreased innate production of IL-6 has also been associated with a decreased risk for developing hand OA, and a trend of decreased risk can be observed for knee and hip OA as well [63]. IL-6 might also be an important link between obesity and OA. It has been shown that infrapatellar fat, and to a lesser extent subcutaneous adipose tissue, can induce the expression of IL-6 in fibroblast-like synoviocytes [64]. Moreover, obese OA patients are known to have higher IL-6 and sIL-6R than non-obese patients with OA [65].

**Figure 4:**
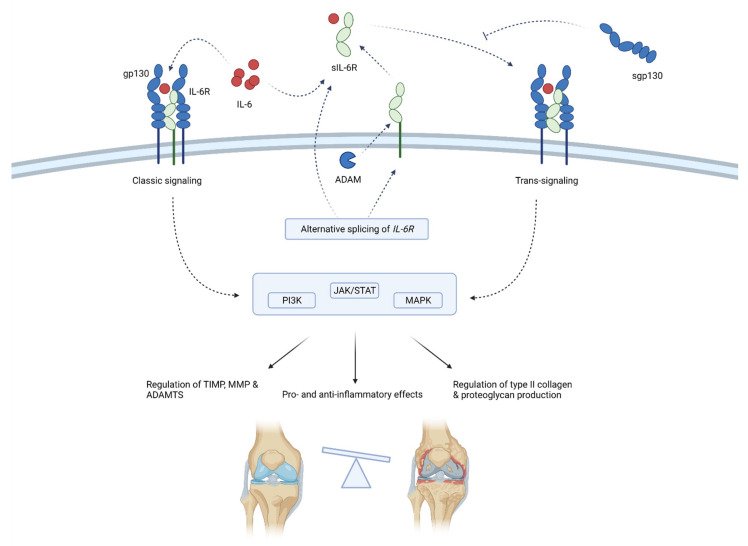
Schematic representation of IL-6 function in osteoarthritis pathogenesis. IL-6 acts by binding membrane-bound IL-6R or sIL-6R that associates with gp130. Gp130 initiates intracellular signaling that regulates the inflammation and expression of enzymes, collagen and proteoglycans. sIL-6R is produced by means of alternative splicing or the shedding of membrane-bound IL-6R. sgp130 can inhibit IL-6 signaling. Through classic and trans-signaling, IL-6 activates the PI3K, JAK/STAT and MAPK signaling pathways that regulate enzymes production (TIMP, MMPs and ADAMTS) and type II collagen and proteoglycan synthesis. Thus, IL-6 balances between anti-inflammatory and proinflammatory effects, but the latter predominates, ultimately leading to the progression of osteoarthritis. ADAM—a disintegrin and metalloproteinase; ADAMTS—a disintegrin-like and metalloproteinase with thrombospondin motifs; gp130—glycoprotein 130; IL-6—interleukin-6; MMP—matrix metalloproteinases; sgp130—soluble glycoprotein 130; sIL-6R—soluble IL-6 receptor; TIMP—tissue inhibitor of metalloproteinase; JAK/STAT—Janus kinase/signal transducers and activators of transcription; PI3K—phosphoinositide 3-kinases; MAPK—mitogen-activated protein kinase.

Thus, it is clear that the interpretation of IL-6 role in OA should not be based solely on the concentration of IL-6 but also on the concentration of sIL-6R and sgp130 and on the assessment of *trans-* and *classic-*IL-6 signaling. This is often overlooked in studies, making it hard to demystify the pathophysiological role of IL-6 in OA. A schematic representation of the mechanism of action and effects of IL-6 is shown in Figure 4.

#### 2.1.4. IL-15

IL-15 is a proinflammatory cytokine produced by various cell types such as fibroblasts, synoviocytes, phagocytes, skeletal muscle, inflammatory cells and many others [66,67]. Although the role of inflammation in the development of the disease has largely been studied in rheumatoid arthritis (RA), recent findings suggest inflammation as an important factor in the pathogenesis of OA. The division of OA into phenotypes speaks in favor of this, defining the inflammatory phenotype of OA among other forms [68]. Likewise, IL-15 has been studied more in the pathogenesis of RA because of its effect on the activation, differentiation and proliferation of T lymphocytes and NK cells [67,69]. However, a study comparing cytokine expressions in synovial fluid of the knee joints of patients with OA and RA showed no significant difference in IL-15 concentrations between OA and RA [70]. Furthermore, according to a study by Scanzello et al., higher concentrations of IL-15 were found in the synovial fluid of patients with early-stage OA compared to patients with late-stage disease, independently of age, gender and BMI [71]. Also, the study found that IL-15 was detectable in all patients with early-stage OA included in the study, indicating its importance in disease progression [71]. Another study found increased IL-15 levels in the serum of patients with OA compared with healthy controls [72,73]. In addition, serum IL-15 correlated with patient-reported pain severity measured by the WOMAC pain score, independently of age, gender and BMI [72].

The exact mechanism by which IL-15 affects the onset and progression of OA is not yet known, but it is known that IL-15 enhances the production of MMPs, as is the case with MMP-9 [74]. It was also shown that the concentration of IL-15 in the synovial fluid of the knee correlates with the concentrations of MMP-1 and MMP-3 and that the concentration of IL-15 in the serum correlates with the concentration of MMP-7 in the serum [71,73]. Despite the association of IL-15 with the enhanced production of matrix-degrading enzymes, an association with the radiological severity of OA has not been established [75]. Precisely because of the unclear mechanism of action, further research is needed to elucidate the role of IL-15 in the development of OA. Elevated concentrations in the early stages of the disease suggest a potential role for IL-15 as a biomarker for the early diagnosis of OA. The confirmation of these findings would be an excellent tool in stopping disease progression in a timely fashion.

#### 2.1.5. IL-17

IL-17, also named IL-17A, is considered a proinflammatory cytokine and a member of the IL-17 cytokine family that includes IL-17B, IL-17C, IL-17D and IL-17E [76]. IL-17A is the most extensively researched cytokine of the IL-17 family, and it has been shown to induce the most potent changes of all IL-17 family cytokines in the transcriptome of synovium and chondrocytes of patients with OA [76,77].

Multiple types of cells secrete IL-17, but little is known about the particular role of these cells in OA and their contribution to IL-17 production in OA. In general, IL-17 is prominently produced by the Th17 subtype of T helper (Th) cells [78]. These cells can act as either pathogenic (responsible for the development of autoinflammatory (AI) diseases, including AI diseases of the joint) or non-pathogenic (protective), depending on the cytokine milieu that stimulates them [78]. The most prominent influence on them is exhibited by IL-23, which is induced in fibroblast-like synoviocytes by IL-17 in RA patients [79]. However, only several studies measured these cells in OA patients, and their explicit role in OA is unclear. Another important source of IL-17 are γδ T cells. These cells are abundant in mucosal tissue and essential in microorganism sensing but also seem to be implicated in bone healing [80,81]. Nevertheless, γδ T cells do not seem altered in the synovial tissue in patients with OA, and we could not find relevant articles investigating γδ T cells’ contribution to IL-17 production in OA [82]. Other known sources of IL-17 include natural killer (NK) cells and macrophages. The abundance of NK cells is present in the synovium and synovial fluid of OA patients [83,84]. Additionally, the enrichment of CD56 ^bright^ CD16 (-) subtypes of NK cells in the synovial fluid, and their numbers, correlate with the concentration of IL-6 [84]. The same cells also accumulate in inflamed tissue and in the interplay with monocytes. They are prone to stimulation by IL-12, IL-15 and IL-18 secreted by monocytes and recurrently stimulate TNF-α secretion in monocytes [85]. These findings accentuate the importance of the inflammatory component to OA, but whether NK cells are a significant source of IL-17 in OA is unknown (unmeasured).

IL-17 signals by binding to the heterodimeric receptors IL17RA and IL17RC to activate downstream NF-κB, MAPK and C/EBP pathways. Although it is a weak activator of NF-κB, IL-17 TRAF-mediated signaling can stabilize the mRNAs of proinflammatory cytokines [86]. IL-17RA and IL-17RC, the main targets for IL-17, are both found on chondrocytes and synovial fibroblasts, with IL-17RA more expressed on the synovial fibroblasts of OA patients with highly inflamed synovium [87]. Furthermore, IL-17 has been found to upregulate catabolic factors (MMP 1, 3 and 13) and downregulate anabolic factors (TIMP3, COL2A1 and SOX9) in chondrocytes isolated from the cartilage of patients with OA and induce cartilage degradation in bovine full depth explant [88,89]. It has also been shown that IL-17 can increase IL-6 and TNF-α production in OA [87]. OA-prone guinea pigs had a higher concentration of IL-17 in comparison to OA-resistant guinea pigs [90]. Moreover, a weak correlation was observed in a longitudinal study of serum cytokines in Hartley guinea pig OA between serum IL-17 and histological score (R2 = 0.16, *p* = 0.047) [91]. Furthermore, a single intra-articular IL-17 injection induces the depletion of proteoglycans with no signs of inflammation, whereas repeated injection induces both inflammation and proteoglycan degradation [92]. 

There are multiple studies with conflicting results investigating IL-17 in the serum and synovial fluid of OA patients. We identified two studies that failed to demonstrate the difference in concentration of IL-17 and the presence of Th17 cells in the serum of patients with OA and the healthy control [93,94]. On the other hand, Qi et al. identified a statistically significant increase in IL-17 concentration in the serum of OA patients. However, the difference between OA and healthy controls is minor (approximately 2 pg/mL, based on Figure 4) [95]. Similarly, Liu et al. have found slightly increased serum IL-17 in OA patients (2.17 pg/mL in control vs. 6.04, 6.35, 6.00 and 5.85 in KL grades 1–4 of OA, respectively). Although not statistically significant, a slight decrease in IL-17 serum concentration is present in KL grade 4 vs. KL grade 1, 2 and 3 [96]. Conversely, another study identified an increase in serum IL-17 only in patients with KL grade 4 OA compared to control (6.161 vs. 4.173 pg/mL), suggesting that IL-17 is increased in patients with more severe OA [97]. We identified two more studies that demonstrated an increase in IL-17 in patients with OA; however, the IL-17 concentration measured in the serum was remarkably higher than in the previous studies (mean values of 106.24 and 134.89 vs. 63.46 and 67.37 pg/mL) [98,99]. Furthermore, IL-17 seems to be negatively associated with infrapatellar fat pat volume and positively associated with the severity of infrapatellar fat pad signal intensity alteration [100]. Both variables are associated with OA, but the observed association with IL-17 is rather tiny. In summation, these results demonstrate high variance, and it is not clear whether IL-17 is altered in OA patients or not. More importantly, the significance of a slight change in the serum IL-17 observed in most of the studies is questionable, especially when changes in the IL-17 target receptors and the other molecules affecting IL-17 signaling are unknown (unmeasured). 

Regarding IL-17 in the synovial fluid, Chen et al. reported that IL-17 concentration from synovial fluid increases from KL grade 2 to KL grade 4, ranging from 5.565 to 8.701 pg/mL, and correlates with the severity of the knee OA graded using the Lequesne index. Although the authors did not specifically comment on this, we observe that the concentration of the IL-17 in the synovial fluid is insignificantly higher than the concentration in the serum [97]. Similarly, two studies reported no difference in the IL-17 measured in the synovial fluid and the peripheral blood [84,101]. A recent study performed measurements of several cytokines in the synovial fluid of patients that underwent total hip or knee arthroplasty, including IL-17, and successfully detected IL-17 in only 14 out of 152 (9.6%). However, the authors suggest that IL-17 identifies an inflammatory OA type based on observed increased IL-6, leptin, resistin, CCL7 and NGF in patients with detectable IL-17 [102]. Similarly, it was reported that the serum IL-17 levels were not significantly associated with cartilage defects and bone marrow lesions, except in a subgroup of patients with hs-CRP of > 2.45 pg/mL, where a moderate association was demonstrated [103].

To date, numerous studies have investigated the association between *IL-17* polymorphism and OA. Lee and Song conducted a meta-analysis and concluded that rs2275913 and rs763780 polymorphism is a risk factor for developing OA [104,105].

#### 2.1.6. IL-18

IL-18, a cytokine primarily identified as an IFN-γ-inducing factor, is a member of the IL-1 family. It is produced, as a biologically inactive precursor (pro-IL-18), by a variety of cells, including chondrocytes, osteoblasts, synoviocytes, macrophages, keratinocytes, dendritic cells, astrocytes, microglia, respiratory epithelial cells and osteoblasts [106,107]. The enzyme responsible for obtaining the active form of IL-18 is caspase-I, known as IL-1 converting enzyme [108].

IL-18 was found in elevated concentrations in the plasma, synovial fluid, and articular cartilage of patients with OA compared with healthy controls, indicating the increased local and systemic production of IL-18 in OA [106]. Furthermore, IL-18 levels, either from plasma, synovial fluid or articular cartilage, were higher in advanced OA than in early-stage OA; that is, they correlated with the radiographic severity of the disease [106]. Furthermore, a more recent study found that synovial fluid IL-18 levels correlated with the severity of post-traumatic OA [109].

IL-18 achieves its effects by various mechanisms of action, which begin when IL-18 binds to its receptors, IL-18Rα and IL-18Rβ, both of which are expressed on chondrocytes [110]. Moreover, IL-18 induces an increase in the number of receptors on the chondrocyte surface and the synthesis of metalloproteinases, MMP-1, MMP-3 and MMP-13, the key enzymes responsible for cartilage degradation [111]. Similar to IL-1β, IL-18 triggers signal transduction via the NF-κB and MAPK-p38-AP1 signaling pathways and thus upregulates COX-2 expression, thereby increasing PGE-2 synthesis in chondrocytes, which inhibits proteoglycan production and aggrecan synthesis but also upregulates MMPs, leading to cartilage degradation [107,112,113,114]. In addition, IL-18 increases the production of NO, a cytotoxic free radical that is an independent factor in cartilage degradation and, unlike the PGE-2 mechanism, is not inhibited by the use of anti-inflammatory drugs [113].

As OA is not a disease of the cartilage but rather of the whole joint, the effects of inflammatory mediators in other tissues within the joint are also important [3]. Thus, COX-2, NO and PGE-2 stimulate both catabolic and anabolic processes in the bone, leading to bone resorption and the formation of osteophytes, respectively [115,116].

Furthermore, IL-18 induces the enhanced expression of genes for the synthesis of IL-6 and TNF-α in chondrocytes and synoviocytes, which further contributes to the fact that one of the most important factors in the pathogenesis of OA is a complex vicious circle of proinflammatory cytokines and other inflammatory mediators [110].

#### 2.1.7. IL-21

Produced by NK cells, Th17 and follicular T-cells, IL-21 is another pleiotropic cytokine involved in immune processes, including OA. The IL-21 signal is transduced when it binds to its receptor (IL-21R) and the common cytokine receptor γ-chain, γ_c_ (shared by the receptors for IL-2, IL-4, IL-7, IL-9 and IL-15), which are found in a variety of cell types [117]. The effect of IL-21 has been thoroughly explored in studies on RA, where its immunologic function is used as a potential drug target [118,119]. In RA, IL-21 levels correlate with IL-17 levels in the sera and synovial fluid of these patients, promoting the production of T_h_17 cells that perpetuate the immune reaction. IL-21 levels also correlate with IL-6 levels, and the inhibition of IL-6 lowers the concentration of IL-21 as well [117]. However, in OA, its place in the underlying immunologic mechanism is still to be defined. In a study by Scanzello et al. IL-21 was found in the majority of synovial fluid samples of patients with cartilage degeneration [71]. A more recent study by Shan et al. increased the levels of IL-21 and IL-21–follicular helper T-cells were found, which correlated with OA severity measured by WOMAC scores and CRP levels, indicating the potential role of IL-21 as a biomarker of OA [120].

#### 2.1.8. IL-22

IL-22 is a member of the IL-10 family produced primarily by Th17 and NK cells. Other cells producing IL-22 include macrophages, neutrophils and fibroblasts [121]. Higher IL-22 concentrations were found In RA and OA joints when there was active synovial inflammation, a common feature of RA but found in OA as well [122]. In OA patients, IL-22 was increased in the synovial fluid and fibroblast-like synoviocytes, and IL-22 receptors were elevated almost tenfold in chondrocytes. Conversely, no difference in IL-22 concentration was observed in the serum of OA patients [123]. Constitutively expressed by fibroblast-like synoviocytes, IL-22 plays an important role in the pathophysiologic mechanism of OA by promoting MMP-1 activity. The potential therapeutic strategy includes blocking this signaling pathway, since it was shown that blocking JAK 2 and JAK 3 decreased the effect of IL-22 on S100A8/A9 [124]. Indeed, in the experimental model of OA, IL-22R neutralizing antibodies proved beneficial [123].

### 2.2. Anti-Inflammatory Cytokines

#### 2.2.1. IL-4

IL-4 is a potent regulator of the immune system and is often called the prototypic immunoregulatory cytokine. It is secreted by Th2 cells, eosinophils, basophils and mast cells [125]. IL-4 is a protein consisting of 129 amino acids, and it takes the form of a four-helix bundle [28].

Its biological effect is achieved by binding to a multimeric receptor system shared with some other cytokines, such as IL-2 and IL-13. There are two different receptor type complexes. Type 1 complex is formed by the dimerization of IL-4Rα and IL-2Rγc and enables the attachment of IL-4, while the interaction between IL-4Rα and IL-13Rα1 forms type 2 complex, which enables the attachment of both IL-4 and IL-13 [28]. The exact signaling pathway of IL-4 is still not clearly described, although there is some relevant information regarding the initial intracellular events. It is known that the gradual phosphorylation of the IL-4Rα/JAK1/STAT3/STAT6 cascade leads to the expression of several proinflammatory genes [126].

There is evidence that polymorphism within the functional candidate gene *IL4R* is associated with OA of the hand, knee and hip [127]. Silvestri et al. found that serum soluble interleukin-4 receptor (sIL-4R) concentration was significantly higher in all OA patients compared to the healthy control group. IL-4 concentration within the synovial fluid and synovial cells was also increased [128,129]. CD4^+^ T-cells were detected in the sublining layer of the synovium of patients with OA, and their number was significantly higher than that of those in the same layer of healthy control. This suggests that the production of IL-4 is primarily determined by T cells (Th2) infiltrating the synovium of the joint [130]. It is worth mentioning that IL-4 has a noticeable chondroprotective effect. It inhibits the secretion of MMPs metalloproteinases, reduces the variation in the production of proteoglycans that are visible in the course of OA and, consequently, has an inhibiting effect on the degradation of proteoglycans in the articular cartilage [131,132]. Furthermore, IL-4 alone or in combination with IL-10 protects against blood-induced cartilage damage and inhibits the apoptosis of both the chondrocytes and FLS [28,133]. Considering its chondroprotective effect and the effect on other cell lineages, it is not surprising that IL-4 decreases the synthesis of inflammatory cytokines such as IL-1β, TNF-α and IL-6 [134]. In addition, IL-4 also decreases the secretion of other inflammatory mediators such as PGE-2, COX-2, PLA2 and iNOS [28].

#### 2.2.2. IL-10

Another cytokine with pleiotropic anti-inflammatory properties is IL-10. IL-10, structurally related to interferons, initiates its effect by binding to its receptor IL-10R—a heterodimer composed of IL-10R1 and IL-10R2 subunits. Mainly produced by immune cells, IL-10 is also synthesized by chondrocytes, where it has a role in the complex mechanism of cartilage extracellular matrix turnover [135]. Upon binding, IL-10R activates the JAK-STAT kinase intracellular pathway and stimulates the expression of genes dependent on IL-10 [28]. The end product of this stimulation is a net chondroprotective, antiapoptotic and anti-inflammatory effect caused by the stimulation of type II collagen and aggrecan synthesis, as well as the inhibition of MMP synthesis [135,136]. Alternatively, IL-10 expresses its profound anti-inflammatory properties by the stimulation of IL-1β antagonist synthesis by macrophages and the inhibition of TNFα, IL-6 and IL-12, thus opposing their proinflammatory effect [28,137]. In vitro IL-10 treatment of cartilage injury model demonstrated a chondroprotective effect and increased glycosaminoglycan content (GAG). Autologous chondrocyte implant grafts treated with IL-10 also demonstrated an improvement in chondrocyte differentiation and cartilage matrix formation [138]. A recent study observed decreased serum levels of IL-10 and the decreased IL-10/TNFα ratio in patients with high-stage knee OA (Kellgren-Lawrence 4) compared with patients with moderate knee OA (Kellgren-Lawrence 3), potentially indicating its prognostic value [139]. The therapeutic effect of physical activity is often taken as an axiom in modern medicine. A clinical study exploring the effect of physical activity on IL levels in 31 female OA patients found increased levels of IL-10 intra- and periarticularly in a 3-h post-exercise period, while IL-6 and IL-8 levels remained stable throughout, thus strengthening the recommendation of physical activity for OA patients [140]. Studies also demonstrated that physical activity promotes M” anti-inflammatory macrophage phenotype differentiation, which in turn produces IL-10 and other anti-inflammatory chemokines and helps in achieving a chondroprotective anabolic joint environment [141]. The effect of mesenchymal stem cell (MSC) therapy on M2 macrophage differentiation has been established as one of the mechanisms by which they stabilize micro-inflammation in knee OA [3,141,142]. Targeted intraarticular plasmid DNA therapy was found to be safe and effective in a canine OA study, highlighting a potential for further treatment options based on IL-10 activity in knee OA [143].

### 2.3. Chemokines

Chemokines, also known as chemotactic cytokines, are small molecules with the ability to induce chemotaxis in a wide variety of cells. They are best known for their effect on the trafficking and guiding of immune effector cells to sites of infection or inflammation. Their wide range of action affects the proliferation, differentiation and activation of cellular responses. Thus, chemokines play an important role in persistent and ongoing inflammation in OA joints [5].

These small (8–12 kDa) protein ligands are divided into four families based on the positioning of the N-terminal cysteine residues: C, CC, CXC and CX3C. In the CC family, the cysteine residues are adjacent to each other. On the contrary, the CXC family is characterized by the separation of the two cysteine residues by an amino acid. The vast majority of known chemokines belong to these two families. The third identified chemokine family is the C family, containing a single cysteine residue in the conserved position. Finally, in the CX3C family, cysteine residues are separated similarly to the CXC family but by three variable amino acids instead of one [144]. Chemokines achieve their effects by binding to G-protein coupled cell-surface receptors. These receptors show different levels of binding specificity and promiscuity, but they do not bind different groups of chemokines. For example, CCR receptors bind only CCL chemokine ligands and CXCR receptors bind CXCL ligands. In order to understand the importance of chemokines in the course of OA, it is inevitable to mention their role in driving cellular motility during the inflammatory response. Leukocytes express a specific set of chemokine receptors and migrate to sites of infection or tissue damage along the gradients of their cognate chemokine ligands. Furthermore, chemokines arrange the recruitment of pluripotent cell types to sites of tissue repair. They perform a variety of functions aside from chemotaxis, including T helper cell differentiation and function as well as angiogenesis, and have a pleiotropic effect on multiple cell types related to the pathogenesis of OA [5,145].

The most important CC family chemokines that are related to OA are CCL2, CCL3, CCL4 and CCL5 [5]. The monocyte chemoattractant protein-1 (MCP-1/CCL2) is a potent chemotactic factor for monocytes that also recruits memory T-lymphocytes and natural killer (NK) cells. Its effects are primarily associated with its binding to the CCR2 receptor [146]. Elevated levels of CCL2 were found in the synovial fluid of patients with both knee injuries and knee OA [147,148]. Miller et al. found that both CCL2 and CCR2 were upregulated in the innervating dorsal root ganglia (DRG) of the knee 8 weeks after surgical injury in a murine model [149]. The same authors did a follow-up study and reported that CCL2 production by murine DRG neurons was induced by alarmin S100A8 and the plasma protein α2 macroglobulin, which are molecular “danger signals” strongly involved in OA pathogenesis [150]. CCL2 (MCP-1) production is dependent on Toll-like receptor-4 (TLR-4) signaling. These findings imply that products of tissue damage and inflammation during OA could stimulate nociceptive pathways. Genetic variation in the CCL2 gene may be associated with knee OA [151]. CCL2 increases MMP-3 expression, which results in proteoglycan loss and the degradation of cartilaginous tissue [152].

CCL3 (MIP-1α), CCL4 (MIP-1β), and CCL5 (RANTES) are other members of the CC family that are also upregulated in OA. Zhao et al. investigated chemokine levels in the plasma of 181 patients (75 control patients, 47 pre-radiographic knee OA patients and 50 radiographic knee OA patients) [153]. CCL3 in plasma showed the highest ability to discriminate pre-radiographic knee OA patients from the control group. Levels in plasma increased with the radiographic severity of the disease. Beekhuizen et al. found that CCL5 levels were among the most significantly elevated mediators in OA synovial fluid compared with controls [60]. Another study that confirms this statement documented CCL5 levels elevation in 18 additional patients [147]. It is worth mentioning that all of these three chemokines are ligands for CCR5. Consequently, Takabe et al. found that CCR5 deficient mice were partially protected against post-traumatic cartilage erosion [154]. There were no signs of bone remodeling or synovial response to surgery, suggesting that CCR5 functions primarily in cartilage during the development of post-traumatic OA. IL-1β-treated human chondrocytes showed the significant upregulation of CCL3, CCL4 and CCL5 [155].

Chemokines from the CXC family that play a significant role in the pathogenesis of OA are CXCL8 (IL-8) and CXCL12. IL-8 is a chemokine molecule, first described as a chemoattractant of neutrophils. Today it is known that IL-8 exhibits effects on many different cells, and it is researched in numerous diseases [156]. It is expressed by cells of the immune system, most prominently CD8^+^ T cells, macrophages and monocytes, but also by keratinocytes, fibroblasts, epithelial cells, hepatocytes and synovial cells [156]. It acts on CXCR1 and CXCR2 receptors expressed not only on leukocytes but also on chondrocytes, osteoclasts, fibroblasts, epithelial and endothelial cells and on the cells of the nervous system [156,157,158].

It has been shown on the human chondrocyte cell line (CHON-002) that IL-8 can be upregulated by TNF-α [159]. Furthermore, IL-8 production is stimulated by advanced glycation end products (AGEs) through NF-κB signaling, which are known to accumulate in cartilage with age and stimulate catabolic metabolism in chondrocytes [160]. Additionally, it has been shown that in human OA chondrocytes, IL-8 is regulated by DNA demethylation that is affected by IL-1b signaling [161]. Free fatty acids also increase the production of IL-8 in the osteoblasts of patients with OA but have little effect on IL-8 secretion in osteoclasts [162]. Osteopontin is yet another molecule involved in the regulation of IL-8 expression, and it is known to stimulate IL-8 in chondrocytes [163]. The mechanical load also increases IL-8 secretion in the chondrocytes of OA patients [164].

Without a doubt, IL-8 is significantly more expressed in the synovial tissue and synovial fluid of patients with RA than in OA [165,166,167,168,169,170]. OA patients undergoing surgery had 37-fold higher IL-8 expression in chondrocytes than patients undergoing surgery due to a fracture of the neck of the femur (likely due to osteoporosis) [161]. Koh et al. have shown that IL-8 is higher in the synovial fluid of OA patients than in young patients with ligament injury [171]. This is also supported by animal studies demonstrating increased IL-8 in dogs with OA [172,173]. Furthermore, it has been shown that IL-8 is also slightly higher in the serum of OA patients than in healthy control [167,171]. IL-8 in synovial fluid has been shown to correlate with the clinical severity of OA, but IL-8 in serum has not [174]. On the other hand, Ruan et al. have demonstrated a certain correlation between serum IL-8 and the clinically and radiologically assessed severity of OA [174,175].

IL-8 is also known to increase collagen I, MMP1- and MMP-13 protein concentration and to enhance the phosphorylation of STAT3 and NF-Kb subunit p65 [159]. It can also affect chondrocyte morphology by decreasing endogenous GTP-Cdc42 and increasing stress fibers. HA concentration in the knee negatively correlates with IL-8 in synovial fluid [170]. In patients with a good response to sodium hyaluronate treatment in terms of improvement of hydrarthrosis, there was a prominent reduction of IL-8 and IL-6 concentration following the treatment [170]. IL-8 also stimulates the hypertrophy of chondrocytes and the calcifications of the matrix [157]. Further studies by the same group have shown that IL-8 increases the expression of PiT-1 expression and stimulates the uptake of inorganic phosphate in chondrocytes [176].

CXCL12, also known as stromal cell-derived factor-1 (SDF-1), is a chemokine that plays a key role in tissue regeneration. It mobilizes mesenchymal stem cells (MSCs) to sites of injury by binding to CXCR4 [177]. Shen et al. confirmed this statement by studying the effects of human meniscus-derived stem/progenitor cells (hMeSPCs) in a rat meniscectomy model [178]. hMeSPCs were injected intra-articularly after meniscectomy and homed to the injured meniscus. The meniscal repair was superior in the hMeSPCs-treated mice, with significantly reduced cartilage degeneration. In a study consisting of 252 patients with knee OA and 144 healthy controls, CXCL12 levels in the synovial fluid were closely related to the radiographic severity of OA [179]. Besides their effect on MSCs, there is evidence that articular chondrocytes express CXCR4, and CXCL12 also induces MMP13 and some other catabolic mediators. The disruption of these catabolic events could be achieved by the pharmacological blockade of CXCL2/CXCR4 signaling. Thus, the disruption of the CXCL12/CXCR4 signaling can be used as a therapeutic approach to attenuate cartilage degeneration in OA [180]. Taking into consideration all of the above, it is obvious that CXCL12 has diverse effects that depend on cellular targets. 

## 3. Conclusions

The pathogenesis of OA is largely determined by the imbalance of proinflammatory and anti-inflammatory mediators, leading to low-grade inflammation, which is responsible for cartilage degradation, bone remodeling and synovial proliferation [181]. Many cytokines, by activating multiple signaling pathways, increase COX-2 expression and consequently PGE-2, which subsequently affects cartilage degradation and osteophyte formation. COX-2 inhibitors such as nonsteroidal anti-inflammatory drugs, which are generally listed as first-line drugs in the relevant guidelines, do not slow down disease progression [182]. The potential answer to this problem lies in the finding that the activation of signaling pathways such as NF-κB and MAPK also activates other mechanisms that lead to OA progression that are not affected by NSAIDs. However, changes in lifestyle habits, such as diet, supplementation and physical activity, can lead to improvements in OA symptoms, even before drug therapies, and it would be of great value to continue research on the mechanism of lifestyle changes on cytokines modulation and slowing OA progression, as it could provide valuable new findings [183,184,185,186]. Furthermore, targeting major cytokines in the development of OA, such as IL-1β and TNF-α, and suppressing their effects did not offer the expected results in clinical studies [18,19,20,187,188]. Since in OA the effects of one cytokine are not necessarily dependent on the activation of another, the suppression of one cytokine may not sufficiently contribute to stopping the inflammation and the production of matrix-degrading enzymes. Therefore, biological treatment methods such as the application of mesenchymal stem cells, which have various anti-inflammatory effects and have obtained significant clinical effects in numerous studies, deserve attention in future research in terms of proving the role of certain anti-inflammatory factors in suppressing OA progression [189,190,191,192,193,194,195]. This potential future research should take into consideration the effect of these therapeutic options on the interplay between different cytokines. Therefore, it would be imprudent to choose a single cytokine to measure the therapeutic effect, as the change of a single variable does not necessarily reflect the change at the level of cellular interactions.

In conclusion, much further research is needed to fully understand the role of cytokines and chemokines in the onset and progression of OA. The epigenetic regulation of cytokine synthesis is also an interesting area to be explored and could offer new solutions in OA management. A sound biological understanding is necessary for any therapeutic intervention to be successful in treating OA patients.
